# Hybrid Processes Combining Photocatalysis and Ceramic Membrane Filtration for Degradation of Humic Acids in Saline Water

**DOI:** 10.3390/membranes6010018

**Published:** 2016-03-01

**Authors:** Lili Song, Bo Zhu, Stephen Gray, Mikel Duke, Shobha Muthukumaran

**Affiliations:** 1 Institute for Sustainability and Innovation, College of Engineering and Science, Victoria University, P.O. Box 14428, Melbourne, VIC 8001, Australia; songll1987@163.com (L.S.); Bo.Zhu@vu.edu.au (B.Z.); Stephen.Gray@vu.edu.au (S.G.); Mikel.Duke@vu.edu.au (M.D.); 2 College of Biological and Environmental Engineering, Zhejiang University of Technology, Hangzhou 310032, China

**Keywords:** ceramic membrane, photocatalysis, humic acid, permeate flux, salinity

## Abstract

This study explored the combined effects of photocatalysis with ceramic membrane filtration for the removal of humic acid in the presence of salt; to simulate saline wastewater conditions. The effects of operating parameters, such as salinity and TiO_2_ concentration on permeate fluxes, total organic carbon (TOC), and UV absorbance removal, were investigated. The interaction between the humic acids and TiO_2_ photocatalyst played an important role in the observed flux change during ceramic membrane filtration. The results for this hybrid system showed that the TOC removal was more than 70% for both without NaCl and with the 500 ppm NaCl concentration, and 62% and 66% for 1000 and 2000 ppm NaCl concentrations. The reduction in UV absorbance was more complete in the absence of NaCl compared to the presence of NaCl. The operation of the integrated photoreactor-ceramic membrane filter over five repeat cycles is described. It can be concluded that the overall removal performance of the hybrid system was influenced by the presence of salts, as salt leads to agglomeration of TiO_2_ particles by suppressing the stabilising effects of electrostatic repulsion and thereby reduces the effective surface contact between the pollutant and the photocatalyst.

## 1. Introduction

Photocatalytic oxidation with UV irradiated TiO_2_ has potential for water and wastewater treatment due to its unique ability for complete mineralization of organic contaminants [1,2,3]. Even though photocatalysts are very efficient for mineralising organic compounds, there are two key challenges. First, effective recovery and recycle of photocatalyst is needed, and second, the by-products formed during the photocatalysis are freely transported into the suspension [4]. Recent studies have explored the separation of TiO_2_ photocatalyst using coagulation and membrane separation [4,5]. When coagulation was used, TiO_2_ particles were flocculated and settled rapidly, but the particles recovered from the sediment required further treatment prior to reuse. Membrane filtration, however, not only aids in the separation of suspended photocatalyst, but may also improve the effluent quality by separation of organic compounds [6]. Therefore, both the above challenges can be resolved using membrane filtration. Moreover, the photocatalytic oxidation process is able to mineralise various organic compounds that are responsible for membrane fouling and thus enhance the consistency of the membrane operation [7].

Most studies have used polymeric microfiltration (MF) and ultrafiltration (UF) membranes to retain powdered TiO_2_ [8,9,10]. However, these membranes still have high fouling tendency when used with TiO_2_ caused by the deposition of contaminants and resulting in a decrease of permeate flux [11]. In work by Lee *et al.*, a photocatalytic membrane reactor was used for the degradation of toxic organic compounds using immobilising TiO_2_ particles on different polymeric membranes and they found that these membranes were slightly damaged by UV irradiation [4]. Other studies showed that the polymeric membranes were damaged by the hydroxyl radicals [12,13] and the penetration of the hard TiO_2_ particles [14]. Current generation ceramic membranes are promising as an alternative to polymeric membranes due to their superior physical integrity, chemical resistance and thermal stability, and in turn have lower chemical demand, lower cleaning frequency and longer lifetime compared to their polymeric counterparts [15,16,17]. The merger of ceramic membranes with advanced oxidation processes is considered a novel and unique opportunity for water treatment [18]. Ceramic membranes can also operate at higher water recoveries, with extended backwash intervals and demonstrate less breakage over their longer life compared to polymeric membranes [16]. There are two major reasons for ceramic membranes to achieve enhanced filtration performance. Firstly, the inclusion of advanced oxidation processes such as ozonation or photocatalytic oxidation, as a pre-treatment step with ceramic membranes can significantly reduce fouling by organic compounds, improve the water quality and in turn reduce the operating cost. Secondly, ceramic membranes offer excellent backwash efficiency as they can withstand high backwash pressure [19].

Generally, wastewaters containing a small concentration of salt and organic compounds are difficult to remove using only conventional filtration or coagulation. Several pre-treatment processes have been adopted before ceramic membrane filtration, such as ozonation and coagulation in order to control fouling in MF/UF membranes [20] and improve the final quality of water [21]. These processes cause the oxidation of organics due to ozonation on the ceramic membrane surface, because of the catalytic properties of metal oxides that form the separation layer of the membrane [22]. Similar findings were reported for treating secondary wastewater using the hybrid ozonation—ceramic membrane filtration [23,24]. For example, Lehman and Liu’s pilot-scale testing on secondary wastewater using combined ozone and coagulation with ceramic membrane filtration [24]. The authors showed that ceramic membrane could operate with minimal fouling at fluxes as high as 212.5 L/m^2^/h. Their long-term ceramic membrane operation at 212.5 L/m^2^/h using 1 mg/L PACl and 4 mg/L ozone pre-treatment demonstrated that little or no fouling occurred and a stable TMP was maintained at 3 psi over a 4-week period. The same studies also highlighted that ozone/coagulation coupled with ceramic membranes offered a potential cost-effective operation due to the ability of operating a very high flux which could offset the more expensive ceramic membrane materials. Therefore the decreasing cost of ceramic membrane coupled with the recent successful development of others hybrid membrane processes like ceramic-ozone system for wastewater applications allow new perspectives for the ceramic membrane-TiO_2_/UV hybrid process.

Recently Benotti *et al.* used TiO_2_/UV and ceramic microfiltration membrane for the removal of pharmaceuticals, endocrine disrupting compounds and estrogenic activity from Colorado River water [25]. It was reported that twenty-nine of the targeted compounds in addition to total estrogenic activity were removed (greater than 70%) while only three compounds were less than 50% removed following the highest level of treatment and no estrogenically active transformation products were formed during treatment. Therefore, ceramic membrane filtration has potential for complete retention of the photocatalyst as well as high molecular weight organic compounds.

The present study explores the effectiveness of combined photocatalytic oxidation and ceramic ultrafiltration of humic acid (HA) in low salinity water. This work is a novel exploration with photocatalysis and ceramic UF membranes as most of the studies have explored polymeric MF membranes. The relative effect of the individual processes featuring in the hybrid system (adsorption, irradiation and membrane filtration) was also assessed for different TiO_2_ and NaCl concentrations. To our knowledge there are no reported studies of this hybrid photocatalytic oxidation and ceramic ultrafiltration for treating saline wastewater. In particular, there is no detailed study on saline wastewater that has salinity of 500–2000 mg/L using this hybrid system.

Humic acid (HA) being a common organic compound in water and wastewater systems, is used as a model compound in this study to assess the performance of this hybrid process. While proteins and polysaccharides are also commonly present in wastewaters, only HA was added in this study to provide a simplified, reproducible experimental system that was easily able to measure removal of HA by TOC and oxidation of HA by UV absorbance at 254 nm (UV_254_). The removal of HA in the presence of salt was considered to simulate saline wastewater conditions and reveal the optimum conditions. More importantly, the combination of photocatalytic oxidation process with physical separation of TiO_2_ via a ceramic membrane filtration was explored. This study is an extension of our previous work where the influence of salinity on the removal of HA using UV/TiO_2_ photocatalytic process alone was examined [26]. The purpose of this study is to evaluate the potential for the hybrid process under ideal conditions prior to exploring on municipal wastewater. The recycling of the spent TiO_2_ slurry for processing fresh feed was also explored. The influences of operating conditions on permeate flux, total organic carbon (TOC) and UV_254_ removal were evaluated. Specifically: (1) the effect of HA and its interaction with TiO_2_ on flux with and without UV irradiation; (2) the effect of salinity on the removal of HA by photocatalytic oxidation and ceramic membrane filtration; (3) the effect of TiO_2_ concentration on flux and HA degradation; and (4) the performance of the combined system with each repeat cycle were investigated.

## 2 Materials and Methods

### 2.1. Materials

HA stock solution was prepared by mixing 6 g of HA powder (Fluka AG Cheische Fabrik., Buchs, Switzerland) in 2 L deionized water, stirred over a period of 2 days with a magnetic stirrer. The stock solution was filtered through a 0.45 μm membrane filter (ADVANTEC, Tokyo, Japan) to remove all suspended solids and stored in a sterilised glass bottle at 4 °C before use. The concentration of the stock solution was 200 mg/L HA based on TOC. The feed solution (HA_0_) containing 20 mg/L HA was prepared by appropriate dilution of stock solution, and resulted in an initial UV absorbance of 0.68 cm^−1^ and TOC concentration of 7 mg/L. Analytical grade chemicals were used without further purification in all experiments. Deionised water was used throughout the experiment.

Titanium dioxide P25 from Evonik (80% anatase, 20% rutile, 99.8% purity, average particle size 30 nm and specific surface area of 50 m^2^/g) was used as the photocatalyst. The TiO_2_ catalyst was obtained as a dry powder and stored at room temperature. Before each set of experiments, the desired amount of TiO_2_ powder was weighed and mixed with a small quantity of deionized water to prepare a TiO_2_ slurry. In general, when nanoparticles are dispersed in liquids, their hydrodynamic size is often larger than the primary particle size [27]. For this system, the particle size of the TiO_2_ (P25) after dispersion with deionized water was measured as 300 nm. However with the addition of HA at pH 7.5, the adsorption of HA on the TiO_2_ surface resulted in an increase in particle size from 300 nm to 500 nm.

### 2.2. Apparatus

**Figure 1:**
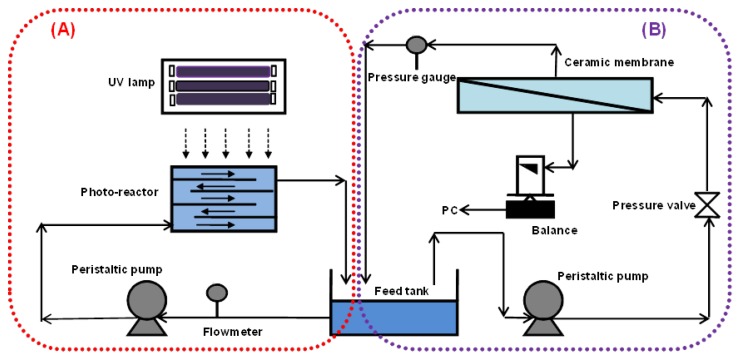
Schematic of a lab-scale photocatalysis/membrane system (**A**) Photocatalytic oxidation system and (**B**) Membrane system.

Figure 1 shows a schematic diagram of the experimental setup, which consists of a photocatalytic reactor (Figure 1A) and a cross-flow ceramic membrane module (Figure 1B). The photocatalytic reactor with a volume of 2 L was made of stainless steel with baffle plates, such that the water flowed in a zig-zag manner through 5 open channels. The total illuminated surface area of the reactor was 713 cm^2^. The UV panel consisted of six 18W UV-A (NEC Blacklight Lamp, Melburne, Australia). UV-A intensity was measured by a UV irradiance meter with a range of 320–400 nm (UV-A, Photoelectric Instrument Factory of Beijing Normal University, Beijing, China).

The membrane used was a tubular titania ceramic membrane (Schumasiv™, Pall Co., Shinjuku-ku, Japan) made of a titania (TiO_2_) coating on an alumina (α-Al_2_O_3_) support with pore size of 5 nm (approximate molecular weight cut off of 10 KDa) and an effective surface area of 48.38 cm^2^. This membrane was chosen for its relative hydrophilicity and ability to reject the TiO_2_ slurry particles given its small pore size. A peristaltic pump (Masterflex 7592-45, Cole-Parmer, Vernon Hills, IL, USA) was used to circulate the suspension at a flow rate of 0.5 L/min in the photocatalytic reactor (Figure 1A) and 1.2 L/min in the ceramic membrane (Figure 1B).

### 2.3. Experimental Procedures

The concentration of HA feed solution (HA_0_) was 20 mg/L for all the experiments. Before each photocatalytic treatment, TiO_2_ slurry and HA were mixed by magnetic stirrer in the dark for 15 min in order to obtain adsorption equilibrium. The solution (2 L), to be treated, was pumped from the feed tank into the photocatalytic reactor at a flow rate of 0.5 L/min and was irradiated by UV for 1 h. Based on the UV irradiation time and hydraulic retention time of the suspension in the reactor, the average exposure time to UV is 20 min. All the experiments were carried out at 20 °C and at a UV intensity of 3.4 mW/cm^2^.

The treated slurry from the batch photoreactor was fed into the ceramic membrane unit for removal and recovery of the TiO_2_ particles. The flux was continuously measured using a balance that recorded the filtrate weight throughout filtration via data acquisition software. All filtration experiments recycled both the retentate and filtrate back to the feed tank. The continuous recirculation of retentate maintained uniform concentration and temperature of the catalyst. Experiments were carried out at a constant TMP of 100 kPa that varied less than 5 kPa throughout the filtration process. Two parameters were varied during this hybrid treatment: NaCl concentration and TiO_2_ concentration, and the effects of these parameters on the permeate fluxes were studied. Most of the experiments were conducted in duplicate.

The membrane was cleaned by soaking in a sodium hydroxide solution (15 g/L) at 85 °C for 30 min prior to each filtration experiment. Following this, the membrane was rinsed with deionized water. The membrane was then soaked in a nitric acid solution (0.1 M) at 50 °C for another 30 min followed by rinsing with deionized water. The effectiveness of the cleaning process was verified by determining the permeate flux through the membranes using deionized water to confirm that the initial membrane flux was the same in all experiments. The permeability of the ceramic membrane was 0.29 L·m^−2^·h^−1^·kPa^−1^.

### 2.4. Selection of Optimum TiO_2_ Concentration

In order to elucidate the role of TiO_2_ catalyst and UV light as well as their combined effects on the HA degradation, experiments were performed for 2 h with different experimental conditions (UV, TiO_2_, and UV/TiO_2_) using an initial HA concentration of 20 mg/L at pH 7.5. Previous research [26] has shown that TiO_2_ concentrations of 0.5 and 1 g/L resulted in significant HA removals of up to 83% using HA solution of 20 mg/L with an initial TOC concentration of 7 mg/L which is similar HA concentration as used in this work. Therefore, these TiO_2_ concentrations were again used in this work.

### 2.5. Analyses

#### 2.5.1. TOC, Turbidity and UV_254_ Measurements

The performance of the hybrid system was monitored through three main output parameters: TOC, turbidity and UV 254 nm absorbance removal. Over the duration of the experiment, 50 mL samples were collected at designated time intervals and filtered through 0.45 μm membrane filters. The TOC concentration of the samples was measured using a Shimadzu TOC V-CSH analyser. The presence of aromatic organic constituents in the water sample was indicated by measuring the absorption of the filtered sample at a wavelength of 254 nm against organic-free water as blank (UV_254_- UV absorbing, Method 10054, HACH). Within the duration of the experiments, the quality of the HA feed solution varied only slightly with an average TOC concentration of 7 ± 0.2 mg/L and the UV absorbance of 0.68 ± 0.2 cm^−1^. The effectiveness of the ceramic membrane process for the separation of photocatalysts was assessed by water turbidity measurements. The turbidity was measured using a HACH 2100 portable turbid meter. The initial suspension and permeate were collected and analysed for turbidity. The result shows that the ceramic membrane is very effective for the separation of TiO_2_ photocatalysts under different NaCl and TiO_2_ concentrations which were less than 0.15 NTU.

#### 2.5.2. Zeta Potential and Particle Size Analysis

The zeta potential and average particle size of the TiO_2_ particles were determined using a Malvern Zetasizer (Malvern Instruments-nano-series). The zeta potential of the TiO_2_ particles were measured from electrophoretic mobility measurements of the particles. During the experiment, samples of suspension containing the TiO_2_ particles and HA solutions were collected regularly in order to analyse the surface charge of the TiO_2_ particles. Control experiments were also conducted by irradiating TiO_2_ suspension in the absence of HA.

#### 2.5.3. Liquid Chromatography (LC)

The apparent molecular weight of the UV adsorbing compounds was determined with LC performed with a photodiode array (PDA) (λ = 200–800 nm) using a TSK gel column (G3000 SW, C-No. SW 3600482) at room temperature with a phosphate buffer (10 mM KH_2_PO_4_ + 10 mM Na_2_HPO_4_, 0.04M, pH 6.8) as the mobile phase. The column was operated with a flow-rate of 0.5 mL/min and a 50 µL injection volume. Polystyrene sulphonate (PSS) molecular weight standards of 3420, 4600, 6200, 15,650 and 39,000 Da were used to calibrate the LC column.

## 3. Results

### 3.1. Effect of TiO_2_/UV Photocatalytic Oxidation of HA on the Permeate Flux

**Figure 2:**
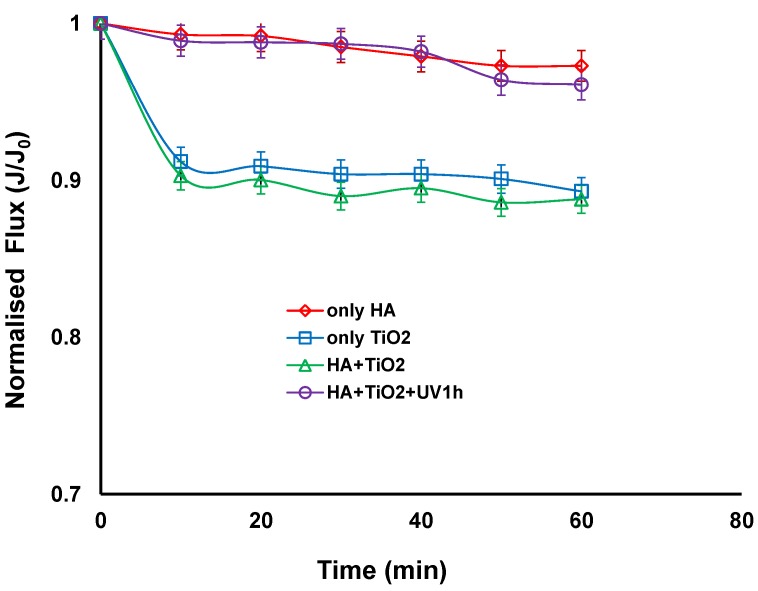
Normalised flux *vs.* time with different combinations of TiO_2_, humic acid (HA), with and without UV irradiation (TiO_2_ concentration: 0.5 g/L, HA concentration: 20 mg/L, Transmembrane pressure (TMP): 100 kPa, Cross-flow Velocity (CFV): 0.4 m/s; pH: 7.5; UV intensity: 3.4 mW/cm^2^).

Figure 2 compares the normalised fluxes for different combinations of feed solutions such as HA alone, TiO_2_ slurry alone, a mixture of HAs and TiO_2_ slurry with and without UV irradiation. When filtration was carried out with HA alone or a combination of HA and TiO_2_ slurry with UV irradiation, the fluxes remained nearly constant. The lack of flux decline with TiO_2_ and UV irradiation reveals that the cake layer of TiO_2_ particles was so porous that it could not provide a noticeable flux decline. This could be associated with the photocatalytic degradation of HA under UV irradiation. Even when HA alone was filtered, no flux decline was observed. Similar results were obtained by Lee *et al.*, where they have found that the lack of flux decline with humic acid when filtering using cellulose acetate UF membrane [4]. They observed a very thin deposition layer of humic acids using scanning electron microscope (SEM) and suggested that humic acids can be deposited or sorbed on the membrane surface during permeation when a solution of humic acids alone was filtered.

When the TiO_2_ slurry alone or TiO_2_ particles were mixed with HA without UV irradiation, there was a slight decrease in the flux (corresponding to a reduction of approximately 10%) during the 60 min of operation. The 300 nm TiO_2_ was much larger than the 5 nm pore size of the membrane. The size of the humic acid has been reported to be in the range of 0.5–30 nm in diameter, which was obtained from the measurement of a molecular weight (MW) distribution by GPC and based on the Einstein-Stokes theory [4]. It can be postulated that a thin cake layer of TiO_2_ particles was formed on the membrane surface when TiO_2_ alone or mixed with HA were filtered. This is consistent with a study by a Xi *et al.* who suggested that HA can be adsorbed onto the surface of TiO_2_ particles and also in the gap between the TiO_2_ particles, resulting in a slight increase of deposition layer resistance, which could be easily removed from the membrane surface by the cross flow velocity of 0.4 m/s [28]. This suggests that HA could fill the pores between the TiO_2_ particles in the cake layer and hence increase resistance, and may also act to bind TiO_2_ particles together requiring higher cross flow velocities to dislodge the TiO_2_ from the membrane surface. For TiO_2_ alone, there would be repulsive forces between particles so this would open the pores in the cake layer and make them easy to remove.

Lee *et al.* however, found a thick cake layer of TiO_2_ particles on a cellulose acetate membrane surface when TiO_2_ and a mixture of TiO_2_ and HA were filtered, and found that the apparent structure of the cake layer of the mixture looked somewhat denser than that of TiO_2_ alone [4]. This was attributed to polymeric membranes adsorbing HA and therefore the HA and HA coated TiO_2_ adhering to the membrane surface and in the cake causing a thick cake layer. A cellulose acetate UF membrane with molecular weight cut off of 30,000 Da was used with a HA concentration of 4 mg/L and TiO_2_ concentration of 0.5 g/L. The use of ceramic membranes in the current study shows that HA solution with and without TiO_2_ slurry did not have a significant effect on the permeate flux, as the potential factors that can affect fouling outcomes such as membrane characteristics and ratio of HA to TiO_2_ are different from their study.

In our study the analysis shows that the cake layer resistance of the HA and TiO_2_ mixed with HA with irradiation deposit on the ceramic ultrafiltration membrane decreased and accounted for only 1.75%% and 2.15% of the total resistance respectively. It was also noticed that the deposit resistance accounted for 9.6% and 10.6% of the total resistance for TiO_2_ alone and TiO_2_ with HA without irradiation respectively. This confirms that the fouling is not severe in all four cases but slightly higher for the TiO_2_ alone and TiO_2_ with HA without irradiation. The results presented above can lead to a conclusion that the influence of TiO_2_ on permeate flux in hybrid system is dual. Firstly, with the presence of TiO_2_, a slight decline of the flux resulting from the formation of a thin cake layer on a membrane surface can take place. Secondly, when the feed solution contains HA, the application of TiO_2_ and UV irradiation can lead to improvement of the flux due to the decomposition of organic molecules by the photocatalytic oxidation process. The lack of flux decline with TiO_2_/UV treatment reveals that the TiO_2_ particles in the cake layer have a wide size and shape distribution which was believed to be closely associated with the change in nature of deposited layers because of the interaction between TiO_2_ particles and the by-products of HA oxidation after UV treatment [28].

### 3.2. Effect of Electrolyte Concentration on the Permeate Flux and HA Degradation

**Figure 3:**
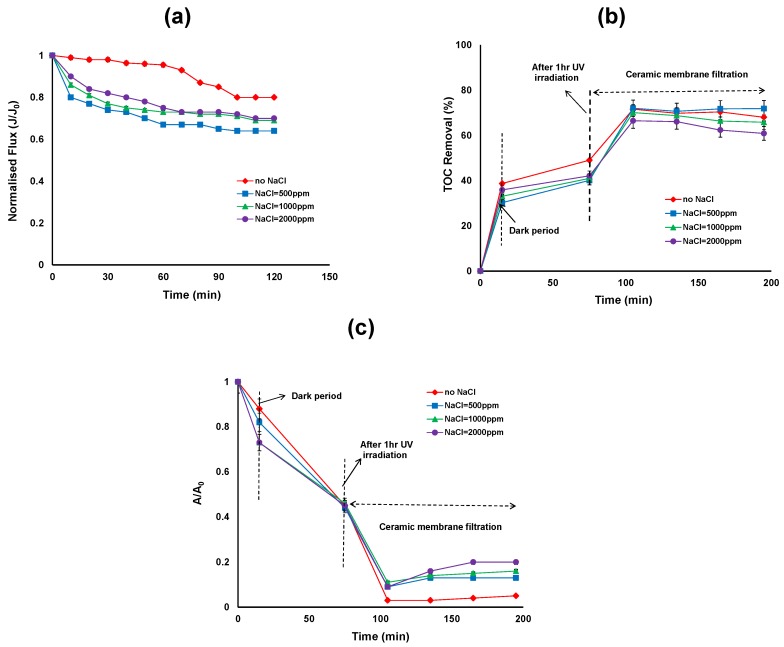
(**a**) Permeate flux (**b**) Total organic carbon (TOC) removal efficiency; (**c**) reduction in UV absorbance of the photocatalytic—ceramic membrane integrated system for various NaCl concentrations (HA concentration: 20 mg/L, TiO_2_ concentration: 0.5 g/L, TMP: 100 kPa, CFV: 0.4 m/s; pH: 7.5; UV intensity: 3.4 mW/cm^2^).

Figure 3a shows the effect of NaCl concentration on the permeate flux after UV/TiO_2_ photocatalytic oxidation reaction. During initial filtration, the flux decline was slightly higher in the presence of NaCl compared to without NaCl. However after 120 min of filtration, the flux reached a steady state in the presence and absence of NaCl and the flux is slightly higher in the absence of NaCl. In the presence of organic matter, TiO_2_ formed larger diameter clusters thereby decreasing the resistance of the TiO_2_ cake layer formed on the membrane surface. The TiO_2_ cluster diameter varied as a function of its zeta potential and thus as a function of pH and electrolyte concentration. Our previous work has shown that the average particle size of the TiO_2_ particle increased and the magnitude of the zeta potential decreased in the presence of NaCl during the photocatalytic treatment [26].

The average hydrodynamic size of TiO_2_ particles in the NaCl suspension was approximately 4000 nm compared to 2900 nm in the absence of NaCl after 120 min photocatalytic treatment. The corresponding zeta potential of TiO_2_ in the presence of NaCl suspension was approximately −9 mV and in the absence of NaCl was −4 mV after 120 min photocatalytic treatment [26]. In the presence of NaCl, the attractive force between particles became dominant over the repulsive force, resulting in an unstable, highly agglomerated dispersion. The energy barrier to prevent agglomeration decreased with increasing ionic strength so the size distributions shift toward larger size ranges with increasing ionic strength. In addition, slightly lower permeate flux was observed at 500 ppm NaCl concentration. Further studies are needed to better understand the effect of lower NaCl concentrations on the flux whereas higher NaCl concentrations (above 500 ppm) have little influence on the flux other than the contribution from the bulk solution properties.

Figure 3b shows that the TOC removal over the duration of hybrid treatment for different NaCl concentrations. The first 15 min of the reaction shows the adsorption of HA on the TiO_2_ surface in the dark, ranging from 39%, 30%, 33% and 37% TOC removal for 0, 500, 1000 and 2000 ppm of NaCl concentrations respectively. According to Huang *et al.* natural organic matter (NOM) adsorption on TiO_2_ was found to be very fast and reached equilibrium in less than 5 min [7]. In our study a 15 min reaction in the dark was sufficient for stable adsorption. The results revealed that without NaCl, HA adsorption on the surface of the TiO_2_ was slightly higher than with NaCl concentrations. After 15 min, UV exposure commenced and after 1 h of UV irradiation, the TOC removal was 49% to 40% for NaCl concentrations between 0 to 2000 ppm. The TOC removal by photocatalysis was low, which suggests the need for higher TiO_2_ concentrations. Further, in the presence of Cl^−^, the TiO_2_ particles in the slurry tend to agglomerate and therefore the surface area available for adsorption of HA and photon absorption decreased. Figure 3c represents the relative UV absorbance removal over the duration of the hybrid treatment. The overall reduction in UV absorbance was more extensive in the absence of NaCl compared to presence of NaCl. However after TiO_2_/UV treatment, no significant reduction in UV absorbance was observed in the presence and absence of NaCl. 

After 2 h of ceramic membrane filtration following photocatalytic oxidation, the TOC removal was more than 70% for 0 ppm NaCl and 500 ppm NaCl concentrations, and 66% and 62% for 1000 and 2000 ppm NaCl concentrations, respectively (Figure 3b). On the other hand, the reduction in UV absorbance was 95%, 87%, 84% and 77% for NaCl concentrations of 0, 500, 1000 and 2000 ppm, respectively (Figure 3c). Results showed that membrane filtration plays a role in the removal of TOC and UV absorbance through the rejection of HA which is either adsorbed on the TiO_2_ particle surfaces or dissolved in the concentrate phase. As the pore size of the ceramic membranes (5 nm) is smaller than the TiO_2_ particles, the TiO_2_ particles were unable to enter into the ceramic membrane pores.

### 3.3. Effect of TiO_2_ Concentration on the Permeate Flux and HA Degradation

**Figure 4:**
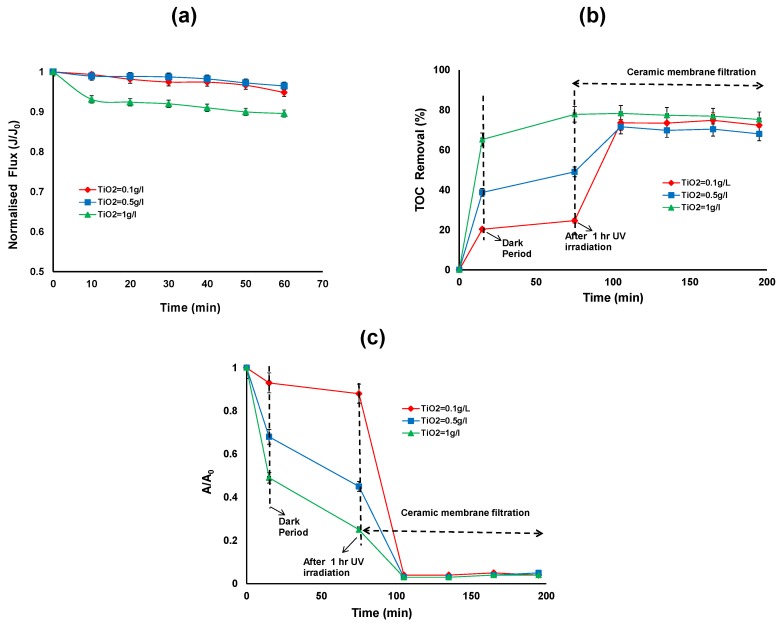
(**a**) Normalised flux (**b**) TOC removal efficiency; (**c**) reduction in UV absorbance of the photocatalytic—ceramic membrane integrated system for various TiO_2_ concentrations (HA concentration: 20 mg/L, TMP: 100 kPa, CFV: 0.4 m/s; pH: 7.5; UV intensity: 3.4 mW/cm^2^).

Figure 4a shows the effect of TiO_2_/UV treatment on the permeate flux for different TiO_2_ concentrations (0.1, 0.5 and 1 g/L). The results indicate that there is no significant permeate flux decline, regardless of the TiO_2_ dose applied. It can be seen that during membrane filtration, the permeate flux was maintained at a level between 26–28 L/m^2^/h for all applied TiO_2_ doses.

Figure 4b,c shows the TOC and relative UV absorbance removal over the duration of the hybrid treatment for different TiO_2_ concentrations. The first 15 min of the reaction shows the adsorption of HA on the TiO_2_ surface in the dark, ranging from 20% to 65% TOC removal for 0.1 to 1 g/L of TiO_2_. After 15 min, the UV exposure commenced and after 1 h of UV irradiation, the TOC removal was 25% to 78% and reduction in UV absorbance was 12% to 75% for 0.1 to 1 g/L of TiO_2_. After 2 h membrane filtration, the TOC removal was more than 70% for all the TiO_2_ concentration (Figure 4b). The TOC removal for HA by the membrane alone was 67%, 30% and 0% for the TiO_2_ concentrations of 0.1, 0.5 and 1 g/L, respectively. It can be observed that at 1 g/L TiO_2_ concentration, there is no further removal of TOC by ceramic membrane filtration. On the other hand the higher removal of TOC by ceramic membrane filtration can be observed at lower TiO_2_ concentrations (0.1 and 0.5 g/L).

The reduction in UV absorbance after 2 h membrane filtration was more than 96% for all the TiO_2_ concentrations. This indicates that the HA was not completely mineralised by photocatalytic oxidation but the aromatic structures of the HA were partially broken or changed to various forms with different adsorptive properties [4].

### 3.4. Recycling of Spent TiO_2_ Slurry

**Figure 5:**
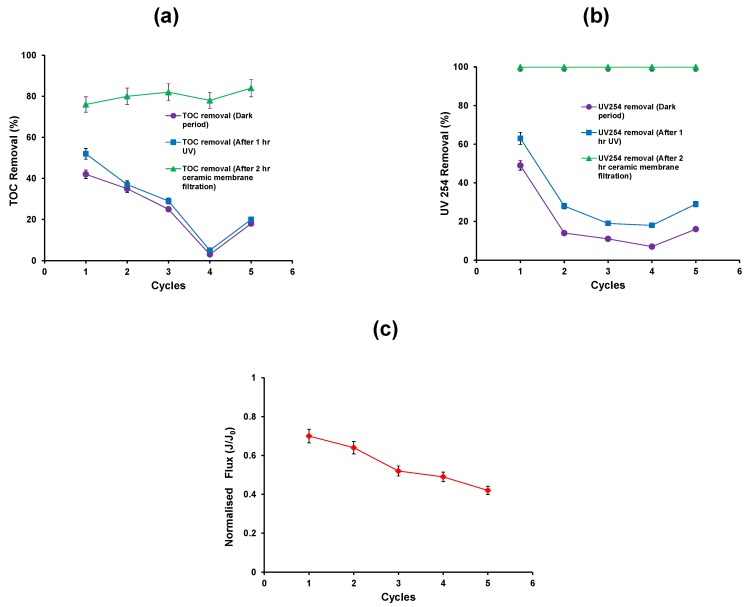
(**a**) TOC removal efficiency; (**b**) reduction in UV absorbance (**c**) Normalised flux of the photocatalytic—ceramic membrane integrated system (HA concentration: 20 mg/L, TiO_2_ concentration: 0.5 g/L, TMP: 100 kPa, CFV: 0.4 m/s; pH: 7.5; UV intensity: 3.4 mW/cm^2^).

Recycling experiments were carried out for five repeat cycles. The experimental procedure was same as discussed in Section 2. The first cycle experiment was carried out in such a way as to obtain 500 mL of concentrate from the ceramic membrane filtration which contained the TiO_2_ suspension. An additional HA solution (1.5 L) was added to this suspension to make 2 L of suspension and this was used as a feed for the second cycle and so on. New TiO_2_ (0.75 g) was only added to make up suspension for the fifth cycle. Figure 5 shows the data from five repeat cycles of operation of the hybrid assembly.

The first 15 min dark equilibration time was chosen such that equilibrium coverage of TiO_2_ particle surface with the HA would have been obtained. The removal of TOC from the adsorption process decreases after the first cycle of operation (Figure 5a). The addition of new TiO_2_ into the system during the fifth cycle shows an increase in the TOC removal during the adsorption process.

A similar trend was also observed for the UV254 removal during the adsorption process (Figure 5b). Following adsorption, the photocatalytic oxidation process was carried out for a period of 1 h. The percent TOC removal and UV 254 removal after the photocatalytic process in the first cycle was 50% and 62%, respectively (Figure 5a,b). Thus the results indicate that the mineralisation of HA was incomplete and the by-products formed would account for the residual organic carbon of the suspension. However, the residual fraction was removed by ceramic membrane filtration such that the total TOC and UV254 removals attained were 76% and 99.9% respectively (Figure 5a,b).

Subsequent cycles of operation show a decrease in TOC and UV254 removal up to the fourth cycle, and increase in the fifth after the addition of fresh TiO_2_. The ceramic membrane filtration results show that the removal of TOC and UV254 was constant for all the cycles. After 2 h membrane filtration, the flux decreases after five repeat cycles as there was no cleaning carried out between the cycles (Figure 5c). It can be postulated that the fouling layer of the TiO_2_ particles during membrane filtration and the consequent re-dispersion stage promote the agglomeration of TiO_2_ particles. This results in a progressive reduction in their photocatalytic activity.

The HA rejection by photocatalytic oxidation processes (both adsorption and oxidation) and ceramic membrane filtration was calculated for all the five cycles. The results show that HA rejection was 25% and 51% by photocatalytic oxidation process and ceramic membrane respectively. HA rejection was decreased from 10% to 1% by photocatalytic oxidation process and increased from 70% to 76% by ceramic membrane filtration from the 2nd to 4th cycle. However, during the 5th cycle *i.e.*, after the addition of new TiO_2_, the HA rejection was slightly increased to 5% and 79% by photocatalytic oxidation process and ceramic membrane filtration respectively. This confirms that the HA is not adsorbed on the TiO_2_ surface and also does not degrade under UV due to the high degree of agglomeration of the TiO_2_ nanoparticles.

Furthermore, the aromatic content per unit concentration of organic carbon SUVA_254_, which is defined as SUVA_254_ = (UV_254_/ TOC) × 100, was determined for both ceramic feed and permeate. The overall SUVA_254_ removal efficiency after ceramic membrane filtration was calculated as the ratio of the difference of the mean feed SUVA_254_ minus the mean permeate SUVA_254_ over the mean feed SUVA_254_. The feed SUVA_254_ value varies between 0.77 and 1.14 m^−1^/(mg/L), whereas the permeate SUVA_254_ is below 0.006 m^−1^/(mg/L) for all the 5 cycles. The overall SUVA_254_ removal efficiency is very high, over 99% in almost all the cycles, furthermore, it is consistently higher than the corresponding TOC overall removal efficiency (Figure 5a). It can be postulated that that the destruction of the aromatic rings in HA molecules, resulting in the reduction of SUVA_254_ values, takes place during the initial stages of HA oxidation, which proceeds in consecutive steps. Therefore partial oxidation of HA macromolecules is possible resulting in oxidation products with reduced aromatic content.

### 3.5. Liquid Chromatography (LC)

**Figure 6:**
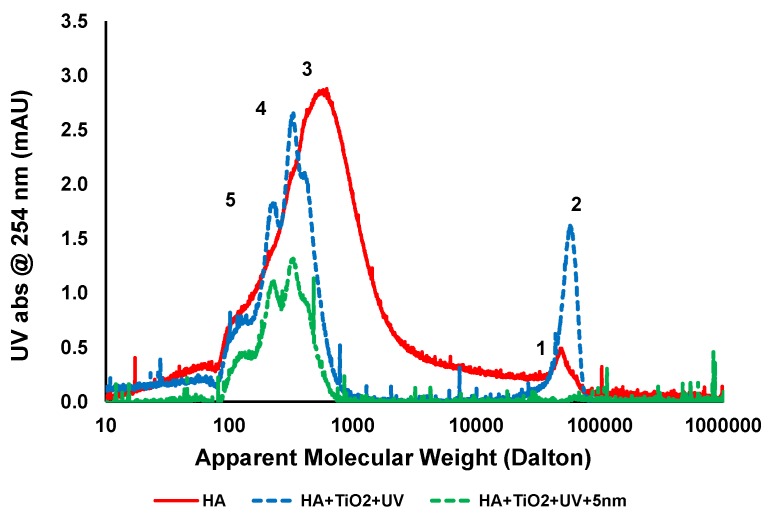
LC analysis of HA at different stages of treatment (HA concentration: 20 mg/L, TiO_2_ concentration: 0.5 g/L, TMP: 100kPa, pH: 7.5; UV intensity: 3.4 mW/cm^2^).

Figure 6 shows LC chromatogram of untreated feed, feed treated with TiO_2_ and UV, and after ceramic membrane filtration. The chromatogram of untreated HA has a broad molecular weight distribution and consists mostly of two main peaks denoted as peak 1 and 3 at molecular weights of approximately 650 and 50,000 Da, respectively. After 1 h UV irradiation with 0.5 g/L TiO_2_ for treatment at pH 7.5, around 50% of peak 3 was removed so the reactions oxidised the organics. Two new peaks denoted as “4” and ”5” corresponding to 340 and 220 Da appeared due to the breakdown of organics into smaller compounds. It has been previously shown that the molecular structures of HA can oxidise and produced two different types of intermediate compounds, having smaller average MW than the peak “3” during the photocatalytic oxidation process [29]. The increase in absorbance intensity of peak “2” from peak “1” of untreated HA sample after 1 h UV irradiation is possibly due to aggregation between small MW compounds and the subsequent degradation of “by-products” which could not be easily removed by TiO_2_ adsorption.

Ceramic membrane filtration showed complete removal of larger molecular weight compounds (peak 2) and up to 50% removal of smaller molecular weight compounds (peaks 4 and 5). In addition the dissolved high molecular compounds were also rejected by membrane filtration and increased the overall performance of the photocatalytic process [28]. Membrane filtration is a purely physical separation process and does not comprise phase change or interphase mass transfer and may be the screening process for a complete recovery of TiO_2_ particles from liquids. As can be seen from the LC analysis, a major portion of the HA contains smaller molecular weight compounds in the range of 100–5000 Da. After TiO_2_/UV treatment, there is a change in the molecular weight distribution of the compounds and preferential adsorption of those compounds by TiO_2_ results in no fouling of the membranes.

## 4. Discussion

Overall, photocatalytic reactions appear to be attractive for the control of membrane fouling and removal of HA during the ceramic membrane filtration of TiO_2_ particles. The results indicate that the TiO_2_/UV treatment reduce membrane fouling, not only through HA adsorption and mineralisation, but also by changing HA molecular characteristics and consequently changing the fouling potential of HA. Further the higher charge density of ceramic surfaces compared to polymer surfaces is likely to increase the significance of electrostatic forces and reduce hydrophobic effects for the adsorption of humic acid on ceramic membrane surfaces [30]. Therefore it is likely that humic adsorption is controlled by electrostatics.

This result shows that the ceramic ultrafiltration membrane with a pore size of 5 nm plays a major role in the removal of TOC and relative UV absorbance even when a low concentration of TiO_2_ was used. Considering the above factors, the proposed mechanism of membrane fouling by HA after photocatalytic treatment is not only by reducing the TOC concentration, but also by changing the molecular characteristics of HA which results from preferential removal of HA fractions responsible for membrane fouling.

The results indicate that the presence of NaCl affects the TOC and UV absorbance removal efficiency during both the photocatalytic and ceramic membrane processes. Generally, the existence of ions may affect the degradation rate via adsorption of the pollutants and reaction with hydroxyl radical ions [31]. A few studies carried out using industrial effluents containing various types of salts which were in ionized form found the effects of different anions and cations on the degradation efficiency [32]. For example, CO_3_^−^, HCO_3_^−^ act as a radical scavengers and also affect the HA adsorption process, while Cl^−^ affects the adsorption and also absorbs UV light. Both the carbonate and chloride ions have strong negative effects on the degradation process compared to anions such as sulphate and nitrate. In this study the results show that the adsorption was slightly impacted in the presence of NaCl and in turn affects the removal efficiency during the photocatalytic treatment. According to Chong *et al.* the presence of salts diminishes the colloidal stability, increases mass transfer and reduces the surface contact between the pollutant and the photocatalyst [33]. Other than fouling of the TiO_2_ surface, chloride ions also scavenge both the hole and the hydroxyl radicals [34]. The mechanism of hole and radical scavenging by chloride has been proposed by Matthews and McEnvoy as follows [33].

(1) $${\text{Cl}}^{-}+{\text{ OH}}^{0}\to {\text{ Cl}}^{0}+{\text{ OH}}^{-}$$

(2) $${\text{Cl}}^{-}+{\text{ H}}^{+}\to {\text{Cl}}^{0}$$

The inhibitory effect of chloride ions occurs through preferential adsorption displacement mechanism which results in reducing the number of OH^−^ available on the photocatalyst surface.

Further, the presence of electrolytes in the suspension causes compaction of the electrostatic double layer around the TiO_2_ particles and decreases the Debye length [28]. This phenomenon can explain the flocculation of TiO_2_ particles and the formation of a particle layer of lower resistance when adding electrolytes. The cations Na^+^, Ca^2+^ and Mg^2+^ present in the waters can bind to TiO_2_ particles, so that they can have significant effects on the interfacial behaviour of the TiO_2_ particles. It is postulated that after photocatalytic oxidation reactions, the smaller molecules of HA adsorbed on the TiO_2_ surface forming HA-Na^+^-TiO_2_ aggregates on the membrane surface result in slightly lower permeate flux compared to without NaCl concentration during the initial period of filtration. The results also indicated that the UV absorbance and TOC removal decrease with salinity during membrane filtration. This may be due to the changes in agglomeration size of the TiO_2_ particles. However the detailed mechanism deserves further investigation. A more thorough chemical composition of these compounds is required for a better understanding of their interactions with the ceramic membranes.

## 5. Conclusions

A hybrid photocatalysis and ceramic membrane system was investigated by treating HA with different saline and TiO_2_ concentrations. The main foci were HA removal and improving permeate flux. The hybrid system results in synergistic effects including oxidation by the photocatalytic reaction, rejection of HA by ceramic membrane filtration and separation of TiO_2_ particles from permeate by membrane rejection. The steady state permeate flux decreases slightly with the presence of NaCl concentrations and the TOC removal, and the reduction in UV absorbance decreased slightly with increasing NaCl concentrations, due to interfacial effects on the ceramic membrane filter. The result indicates that the presence of NaCl affects the removal efficiency during both the photocatalytic and ceramic membrane processes.

Ceramic membrane filtration ensured the complete separation of the photocatalyst from treated suspensions under different TiO_2_ and NaCl concentrations. Degradation in the performance of the photocatalytic system with each repeat cycle was observed in this study. On the other hand, ceramic membrane filtration maintained consistently high removal across all the five repeat cycles. A reduction in membrane fouling is recognised to arise mostly from a change in HA molecular characteristics resulting from preferential removal of high molecular weight HA molecules that are major contributors to membrane fouling. Considering the removal of HA fouling potential, the relatively high TOC and UV absorbance removal and the complete recovery of TiO_2_ slurry, the hybrid process could be a very promising method for water and wastewater treatment.
